# TOFF, a database of traits of fish to promote advances in fish aquaculture

**DOI:** 10.1038/s41597-019-0307-z

**Published:** 2019-12-03

**Authors:** Thomas Lecocq, Alain Benard, Alain Pasquet, Sarah Nahon, Alex Ducret, Kevin Dupont-Marin, Iris Lang, Marielle Thomas

**Affiliations:** 1 University of Lorraine, INRA, Research Unit Animal and Functionalities of Animal Products (URAFPA), 54000 Nancy, France; 2 INRA - AgroParisTech - U. Lorraine, UMR 1434 SILVA. Centre de recherche Grand Est-Nancy, 54113 Champenoux, France; 3 CNRS (National Centre for Scientific Research), DR6 - 54500 Vandoeuvre-lès-Nancy, France; 4 INRA, Université de Pau et des Pays de l’Adour, E2S UPPA, UMR 1419, Nutrition, Métabolisme, Aquaculture, Saint Pée sur Nivelle, F-64310 France

**Keywords:** Ichthyology, Agroecology

## Abstract

Functional traits can be valuable pieces of information for aquaculture research and management. Although fish traits have been the focus of an abundant research, trait datasets for these organisms are difficult to access and often unpractical to achieve meta-analyses without a time-consuming extensive review. Already available large-scale compilations include trait information for many fish species but not as detailed as required for aquaculture purpose. Here, we introduce the TOFF (i.e. Traits OF Fish), a database focusing on fish functional traits that aims at bringing together behavioral, morphological, phenological, and physiological traits always coupled to environmental measurement context into a single open-source access repository. TOFF hosts data from published field and experimental studies. Here, we release data for 228 traits for 174 species extracted from 165 publications and present a collaborative platform. We ultimately aim at providing an inclusive and accessible data resource to facilitate advances in aquaculture development.

## Abstract (summary)

**Table:** 

Measurement(s)	behavior • morphology • phenological trait • physiological trait
Technology Type(s)	digital curation
Factor Type(s)	taxonomy • ploidy level • sex • developmental stage
Sample Characteristic - Organism	Myxiniformes • Hyperoartia • Holocephali • Elasmobranchii • Sarcopterygii • Actinopterygii

Machine-accessible metadata file describing the reported data: 10.6084/m9.figshare.10276040

## Background & Summary

Aquaculture is the farming of aquatic organisms in coastal or inland areas involving human control over the rearing/breeding process to enhance production^1,2^. Capture stagnation in wild fisheries and overexploitation of popular consumed species along with an increasing human demand for aquatic products have triggered an unprecedented development of aquaculture since the 1960s^1–3^. Nowadays, aquaculture provides about 50% of the world’s aquatic food consumption and still continues its exponential growth^4^. The worsening biodiversity crisis of aquatic species (e.g., for fishes^5,6^) and international organization recommendations (e.g.,^7^) are further encouraging aquaculturists to diversify their species production. However, producing new species is challenging because it requires to rear/breed organisms in an optimal way (e.g., efficient feeding practices, cost-effective captive environment) in human-controlled environment (e.g., ponds, netted cages, or re-circulated aquaculture systems in monoculture or polyculture context)^2^ on a long-term basis despite potential disturbances (e.g., climate change^7^). This challenge cannot be achieved without detailed knowledge of species biology and ecology^2^.

Functional traits are any phenotypic characteristic of individual organisms that impacts (in)directly the fitness of such organisms^8^ and is relevant to their effects on ecosystem properties^9^. These traits include behavioral, morphological, phenological, and physiological traits (BMPP traits) important to know how organisms interact with their environment and with other species^8,10^. Although this paradigm has not been widely applied to agricultural research and management, the functional trait approach can be applied to agro(eco)systems by understanding the role of agrobiodiversity (i.e. species composition and structure) in ecosystem processes and services^11,12^. This allows understanding or predicting interactions and fitness of species in a farmed environment to ultimately enhance the efficiency and sustainability of agriculture^12^, including aquaculture^13^. For instance, estimating feasibility and efficiency of new species combination by functional trait-based approach is a useful tool to promote polyculture practices which aim at culturing species displaying complementary resource uses (i.e. minimizing aquafarming environmental impact and maximizing production profitability) and minimal resource competition (see polyculture advantages in^14^). Similarly, functional trait analyses allow projecting the consequences of cultured environment modifications such as those triggered by climate changes on species production. Therefore, functional trait-based approach can limit time and money-consuming trial-and-error bioassays to assess alternative aquaculture development scenarios. This places a premium on the compilation of species trait datasets with broad taxonomic coverage in species groups significant for aquaculture, such as fishes, to foster applied developments through big data-based meta-analyses.

For more than a century, an abundant research has extensively studied the fish traits for a variety of purposes. This means that sufficient data might well already exist for addressing alternative development hypotheses and meta-analyses about fish aquaculture. However, an overview of broad-scale insights is poorly available because useful datasets are scattered over several decades of literature in many languages. Some trait compilations have already been proposed (e.g.,^15–18^) but most of them focus on particular set of traits to address specific questions, fail to ensure homogeneity of data (e.g., no standardized definitions of traits making comparison from different sources doubtful), gather information for a limited geographic area/taxonomic range, and/or has restricted availability. Although original citations are provided, the largest available comprehensive compilations of fish traits (i.e. FishBase [www.fishbase.org] or^19^) tend to provide only a synthesized mixture of original and meta-analysis datasets. Furthermore, the lack of important contextual information about measurement environmental context or levels of variation and replication for trait data limits such compilations. Yet, functional traits are environment-dependent^20^ and display variability at the interpopulational level^21^. Therefore, a database hosting detailed information about functional traits and their environmental measurement context, which are required for aquaculture purpose, is still lacking.

Here, we introduce TOFF (i.e. Traits OF Fish) database, the first curated collaborative database of functional trait information for fish species significant for aquaculture (i.e. seawater, brackish, and freshwater species). The aims of the database are: (i) to gather disparate information on fish traits associated with information about measurement environmental context, (ii) to provide unrestricted open-source access to fish trait data, and (iii) to engage the fish research community in the collection and quality control of trait datasets. We selected 228 validated, defined, and referenced BMPP traits with their measurement environmental context (i.e. 108 environmental features). The database can be fed, consulted, and modified by the fish research/aquaculture community through an online system archiving and presenting fish trait data for future research. We ultimately aim at providing an inclusive and accessible data resource to facilitate new advances in aquaculture.

## Methods

TOFF focuses on functional traits of fish (*sensu* all taxa included in Myxini [hagfishes], Cephalaspidomorphi [lampreys], Holocephali [chimaeras], Elasmobranchii [sharks and rays], Sarcopterygii [lobe-finned fishes], and Actinopterygii [ray-finned fishes]), the most taxonomically diversified group among chordates with more than 34,000 species (according to FishBase [www.fishbase.org]). The database can host various types of data from multiple sources of literature (i.e. peer-reviewed articles, theses, monographs, and books). It has been designed to contain “records” that are information about BMPP traits for a fish species, at a specified developmental stage (i.e. larvae, juvenile, or adult), sex, and ploidy level in a characterized environment from a scientific source. This means that a study considering, for instance, different environments and/or developmental stages, sexes, and ploidy level is encoded as several records in the database. A record can be based on individual or group measurements from field observations or *ex-situ* experiments.

### Database description

Considering the heterogeneous formats and contents of data referring to BMPP traits and environment measurements, we develop a flexible SQL relational database using EAV (entity - attribute - value) model coupled with controlled vocabularies. These latter ones are semantic tools, which consist of a list of terms, their definitions, and some of their properties such as their unit, their range (e.g., from 0 to 14 for pH), their spelling into drop-down menu (e.g., for categorical variables), or their format (see detailed thesaurus in^22^). Automatic controls are implemented in the database following the format, the spelling, and/or the range following thesaurus guidelines. TOFF uses postgreSQL as database management system. Each record in the database is unique, identifiable, checked by scientific expert board, and traceable. The database is hosted by the data center of *the Institut national de la recherche agronomique* (INRA) and baked up daily.

**Fig. 1:**
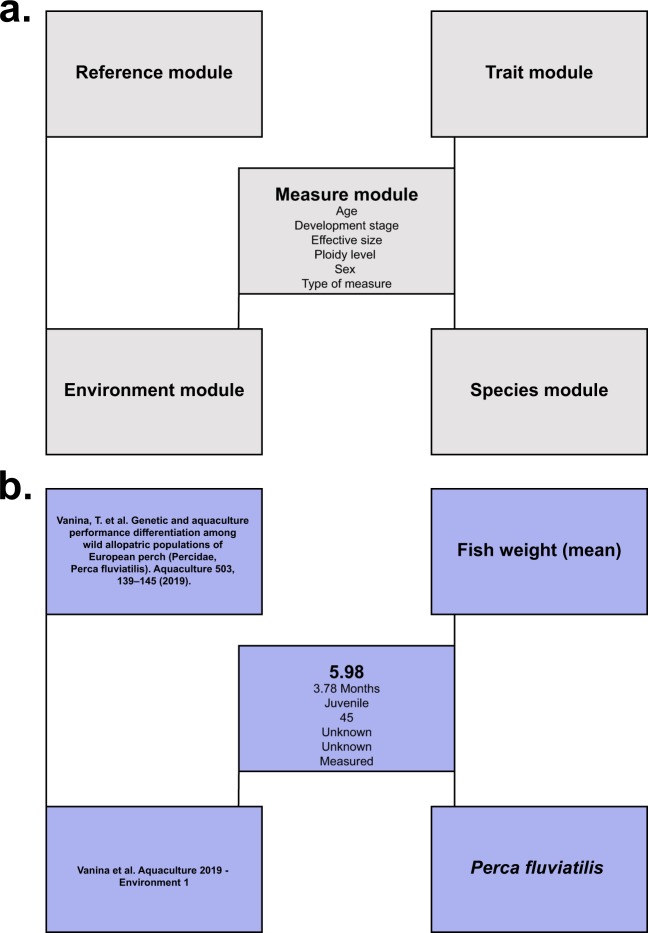
Overview of the design of the TOFF database. (**a**) General scheme of the database. (**b**) An example (from^24^) of a trait measure integrated in this scheme.

The relational database schema contains five core modules (Fig. 1, see detailed data model in^22^). First, the Reference module stores bibliographic metadata (e.g., authorship, publication type, publication year). Second, the Trait module integrates functional traits included in the database. These traits are grouped into four classes (i.e. behavioral, morphological, phenological, and physiological traits) and defined following the thesaurus^22^. Third, the Measure module includes each measure (i.e. attribute of a trait) of a particular trait. It is linked to the Trait module. The Measure module includes also information about the effective size (i.e. notifying on how many individuals is based the trait measure), the ploidy level (e.g., diploid or triploid fish), the development stage (i.e. larvae, juvenile, adult, or unknown), the sex (i.e. male, female, hermaphrodite, or unknown), the type of measure (i.e. detailing if the trait measure is based on a direct measurement, computed values, or predicted through modeling approach), and the name of measure recorder (i.e. the data contributor for a particular measure) associated to each measure. Fourth, the Environment module displays information about the environment in which the measures have been recorded. The environmental features are classified into three classes: abiotic environments (e.g., geographic occurrence, water parameters), biotic environment (e.g., population density, environment species diversity), and information about feeding (e.g., *ad libitum*). Fifth, the Species module (i.e. providing a table with the valid names of fish species based on FishBase species database (www.fishbase.org) extracted through the R-package rfishbase [current extraction date: January 2017]; this table is regularly updated to integrate taxonomic revision).

### Data acquisition

All records in the TOFF database are extracted from (i) published resources including peer-reviewed articles, theses, monographs, and books or (ii) experimental studies (unpublished data). In the former case, the original source is always specified and must be provided by contributors. This allows performing meta-analyses citing all relevant references and integrating some or all type of published resources. Three types of data are recorded: continuous numerical values, discrete, or categorical data. Measurement value types include mean, standard deviation, median, maximum, minimum, and model derived. Continuous data are typically means extracted from tables or figures unless raw data are available. Aggregate values (e.g., means and medians) are always accompanied by the number of replicates and a measure of dispersion (e.g., standard deviation). The dataset here released have broad taxonomic range. However, some large data gaps exist because of the time-consuming review process required to feed the database. This places a premium on the swift development of a contributor community for TOFF to increase the dataset included in the database. In order to facilitate such a development, we propose an optimized importation tool from csv files to integrate large dataset in TOFF (see TOFF Assistant website: http://toff-project.univ-lorraine.fr).

## Data Records

The thesaurus and detailed data model of TOFF are provided on figshare^22^ as a xlsx file and a pdf file, respectively. The current static release of TOFF contains 5143 records informing on 228 BMPP traits for 174 species from 165 references. The first steps of encoding work have focused on species with economical interest for freshwater aquaculture and their secondary/subordinate taxa. The static release as two csv files is available on figshare^23^. Details and references were produced with the R-script file provided on figshare^23^. Up-to-date database can be downloaded directly from this R-script file.

## Technical Validation

TOFF is managed on a voluntary basis by an editorial board and database administrators. We develop a four-stage quality control of data and managing procedure. First, people willing (i.e. new contributor access) to be database contributors (or to be member of editorial board) must notify their demand to the editorial board through the TOFF website (http://toff-project.univ-lorraine.fr). They will receive an account and an access to the database through on-line system. Second, new data added by database contributors are not directly integrated to the main database but store in temporary repository. The integration of such new data in TOFF requires a prerequisite assessment by the editorial board (i.e. editor approval). Third, data issues noted by users can be reported to the editorial board through the TOFF website (i.e. user feedback). Fourth, in order to avoid duplicate, measurements with the same value, reference, location, and species will be automatically detected during the integration of database contributors to the main database.

### Further developments

Currently, TOFF contains traits of only a fraction of fish species (e.g., lacking marine species) compare to the large number of species and thus some taxonomic classes are completely absent. Indeed, developing an extensive database is a long and challenging endeavor difficult for only few researchers. Therefore, TOFF is designed as a collaborative platform to integrate a large group of researchers worldwide in TOFF further developments. This means that the taxonomic coverages, the functional trait list, the relevant environmental descriptors, and the editor board will evolve over time thanks to contributions of interested researchers.

## Usage Notes

The data release is available on figshare^23^. This release was produced by an R-script available for TOFF users on figshare^23^.

## References

[1] Food and Agriculture Organization of the United Nations. *The State of World Fisheries and Aquaculture 2018 - Meeting the sustainable development goals*. (Food and Agriculture Organization of the United Nations, 2018).

[2] Teletchea F, Fontaine P (2014). Levels of domestication in fish: implications for the sustainable future of aquaculture. Fish Fish..

[3] Ahmed N, Thompson S (2019). The blue dimensions of aquaculture: A global synthesis. Sci. Total Environ..

[4] Food and Agriculture Organization of the United Nations. *The State of World Fisheries and Aquaculture 2016. Contributing to food security and nutrition for all*. (Food and Agriculture Organization of the United Nations, 2016).

[5] Dudgeon D (2006). Freshwater biodiversity: importance, threats, status and conservation challenges. Biol. Rev. Camb. Philos. Soc..

[6] Nieto, A. *et al*. *European Red List of marine fishes*. (Publications Office of the European Union, 2015).

[7] Food and Agriculture Organization of the United Nations. *Planning for aquaculture diversification: the importance of climate change and other drivers*. (Food and Agriculture Organization of the United Nations, 2017).

[8] Violle C (2007). Let the concept of trait be functional!. Oikos.

[9] Díaz S (2013). Functional traits, the phylogeny of function, and ecosystem service vulnerability. Ecol. Evol..

[10] Díaz S, Cabido M (2001). Vive la différence: plant functional diversity matters to ecosystem processes. Trends Ecol. Evol..

[11] Wood SA (2015). Functional traits in agriculture: agrobiodiversity and ecosystem services. Trends Ecol. Evol..

[12] Martin AR, Isaac ME (2015). Plant functional traits in agroecosystems: a blueprint for research. J. Appl. Ecol..

[13] Costa-Pierce, B. A. In Sustainable *Fo*od Pr*oduction. Spring*er (eds. Christou, P., Savin, R., Costa-Pierce, B. A., Misztal, I. & Whitelaw, C. B. A.) 174–183 (Springer New York, 2013).

[14] Stickney, R. R. In *Sustainable* Food *Production* (eds. Christou, P., Savin, R., Costa-Pierce, B. A., Misztal, I. & Whitelaw, C. B. A.) 1366–1368 (Springer New York, 2013).

[15] Teletchea F (2007). STOREFISH: A new database dedicated to the reproduction of temperate freshwater teleost fishes. Cybium.

[16] Blanck A, Lamouroux N (2007). Large-scale intraspecific variation in life-history traits of European freshwater fish. J. Biogeogr..

[17] Mims MC, Olden JD, Shattuck ZR, Poff NL (2010). Life history trait diversity of native freshwater fishes in North America. Ecol. Freshw. Fish.

[18] Côte J, Kuczynski L, Grenouillet G (2019). Spatial patterns and determinants of trait dispersion in freshwater fish assemblages across. Europe. Glob. Ecol. Biogeogr..

[19] Frimpong EA, Angermeier PL (2009). Fish Traits: A Database of Ecological and Life-history Traits of Freshwater Fishes of the United States. Fisheries.

[20] Ghalambor CK, Mckay JK, Carroll SP, Reznick DN (2007). Adaptive versus non-adaptive phenotypic plasticity and the potential for contemporary adaptation in new environments. Funct. Ecol..

[21] Alofs KM (2016). The influence of variability in species trait data on community-level ecological prediction and inference. Ecol. Evol..

[22] Lecocq T (2019). figshare.

[23] Lecocq T (2019). figshare.

[24] Vanina T (2019). Genetic and aquaculture performance differentiation among wild allopatric populations of European perch (Percidae, *Perca fluviatilis*). Aquaculture.

